# The genome sequence of the sardine, *Sardina pilchardus *(Walbaum, 1792)

**DOI:** 10.12688/wellcomeopenres.22826.2

**Published:** 2024-11-04

**Authors:** Rachel Brittain, Patrick Adkins, Kesella Scott-Somme, Joanna Harley, Vengamanaidu Modepali, Marine Biological Association Genome Acquisition Lab, Darwin Tree of Life Barcoding collective, Wellcome Sanger Institute Tree of Life Management, Samples and Laboratory team, Wellcome Sanger Institute Scientific Operations: Sequencing Operations, Wellcome Sanger Institute Tree of Life Core Informatics team, Tree of Life Core Informatics collective, Darwin Tree of Life Consortium

**Affiliations:** 1 The Marine Biological Association, Plymouth, England, UK

**Keywords:** Sardina pilchardus, sardine, genome sequence, chromosomal, Clupeiformes

## Abstract

We present a genome assembly from an individual *Sardina pilchardus* (the sardine; Chordata; Actinopteri; Clupeiformes; Clupeidae). The genome sequence spans 869.40 megabases. Most of the assembly is scaffolded into 24 chromosomal pseudomolecules. The mitochondrial genome has also been assembled and is 17.57 kilobases in length.

## Species taxonomy

Eukaryota; Opisthokonta; Metazoa; Eumetazoa; Bilateria; Deuterostomia; Chordata; Craniata; Vertebrata; Gnathostomata; Teleostomi; Euteleostomi; Actinopterygii; Actinopteri; Neopterygii; Teleostei; Osteoglossocephalai; Clupeocephala; Otomorpha; Clupei; Clupeiformes; Clupeoidei; Clupeidae; Clupeinae; *Sardina*; *Sardina pilchardus* (Walbaum, 1792) (NCBI:txid27697).

## Background

The European sardine ( *Sardina*, or *Clupea pilchardus*) also commonly referred to as pilchard in Britain ( Artedi *et al.*, 1792), is a small pelagic fish found in temperate boundary currents of the Northeast Atlantic down to Cape Verde off the west coast of Africa, and throughout the Mediterranean to the Black Sea ( Grant *et al.*, 2014; Parrish *et al.*, 1989). Sardines are part of a diverse taxonomic group that also includes marine or freshwater pelagic fish such as shad and herring. In comparison to herring, sardines are smaller, with an average length of just over 20 cm. They usually have a lifespan of up to ten years and tend to spawn mostly in the spring ( Coombs *et al.*, 2006). Eggs, larvae, and juveniles are all pelagic. Sardines are usually microphagous filter feeders (zooplankton and/or phytoplankton), but the type of food ingested varies with age. Although *Sardina pilchardus* is capable of living at a depth of 180 m, it usually stays at shallower depths of 35 to 55 m during the day and 13 to 35 m at night ( Fischer & Food and Agriculture Organization of the United Nations, 1973).

*Sardina pilchardus* is of high economic importance throughout its distribution. The sardine experiences strong population fluctuations in abundance, possibly reflecting environmental changes ( Checkley *et al.*, 2017). Along with climatic changes overfishing exacerbates the issue ( Leitão *et al.*, 2014). Small pelagic species like *Sardina pilchardus* account for about a quarter of the global fish catch, making them both economically and ecologically significant.

The *Sardina pilchardus* genome will aid in assessing the genetic foundation of the sardine population. It will also be useful for genetic research on the life history and ecological characteristics of this small pelagic fish. This information will be crucial for effective conservation and fisheries management.

## Genome sequence report

**Figure 1.**
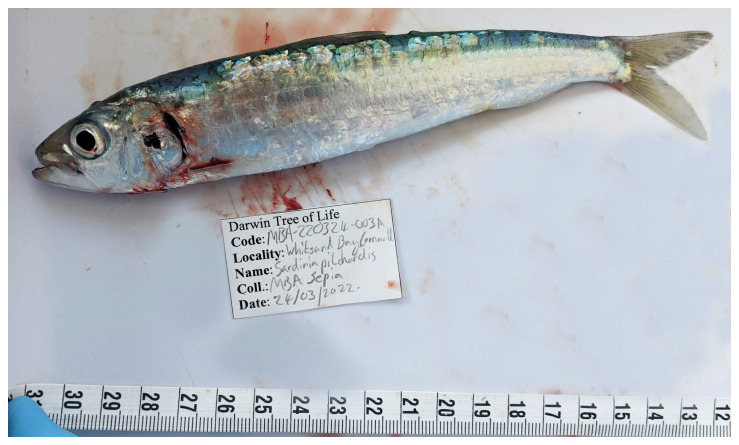
Photograph of the *Sardina pilchardus* (fSarPil1) specimen used for genome sequencing.

The genome of an adult *Sardina pilchardus* of unknown sex ( Figure 1) was sequenced using Pacific Biosciences single-molecule HiFi long reads, generating a total of 46.88 Gb (gigabases) from 4.62 million reads, providing an estimated 33-fold coverage. Primary assembly contigs were scaffolded with chromosome conformation Hi-C data, which produced 80.39 Gb from 532.38 million reads, yielding an approximate coverage of 92-fold. Specimen and sequencing information is summarised in Table 1.

**Table 1.** Specimen and sequencing data for *Sardina pilchardus*.

Project information			
**Study title**	Sardina pilchardus (sardine)		
**Umbrella BioProject**	PRJEB67417		
**Species**	*Sardina pilchardus*		
**BioSample**	SAMEA111562172		
**NCBI taxonomy ID**	27697		
Specimen information			
**Technology**	**ToLID**	**BioSample ** **accession**	**Organism part**
**PacBio long read** **sequencing**	fSarPil1	SAMEA111562362	gill
**Hi-C sequencing**	fSarPil1	SAMEA111562362	gill
**RNA sequencing**	fSarPil1	SAMEA111562362	gill
Sequencing information			
**Platform**	**Run accession**	**Read count**	**Base count (Gb)**
**Hi-C Illumina NovaSeq 6000**	ERR12121868	5.32e+08	80.39
**PacBio Sequel IIe**	ERR12120045	1.73e+06	19.7
**PacBio Revio**	ERR12120044	2.89e+06	27.18
**RNA Illumina NovaSeq 6000**	ERR12121869	5.69e+07	8.6

**Figure 2.**
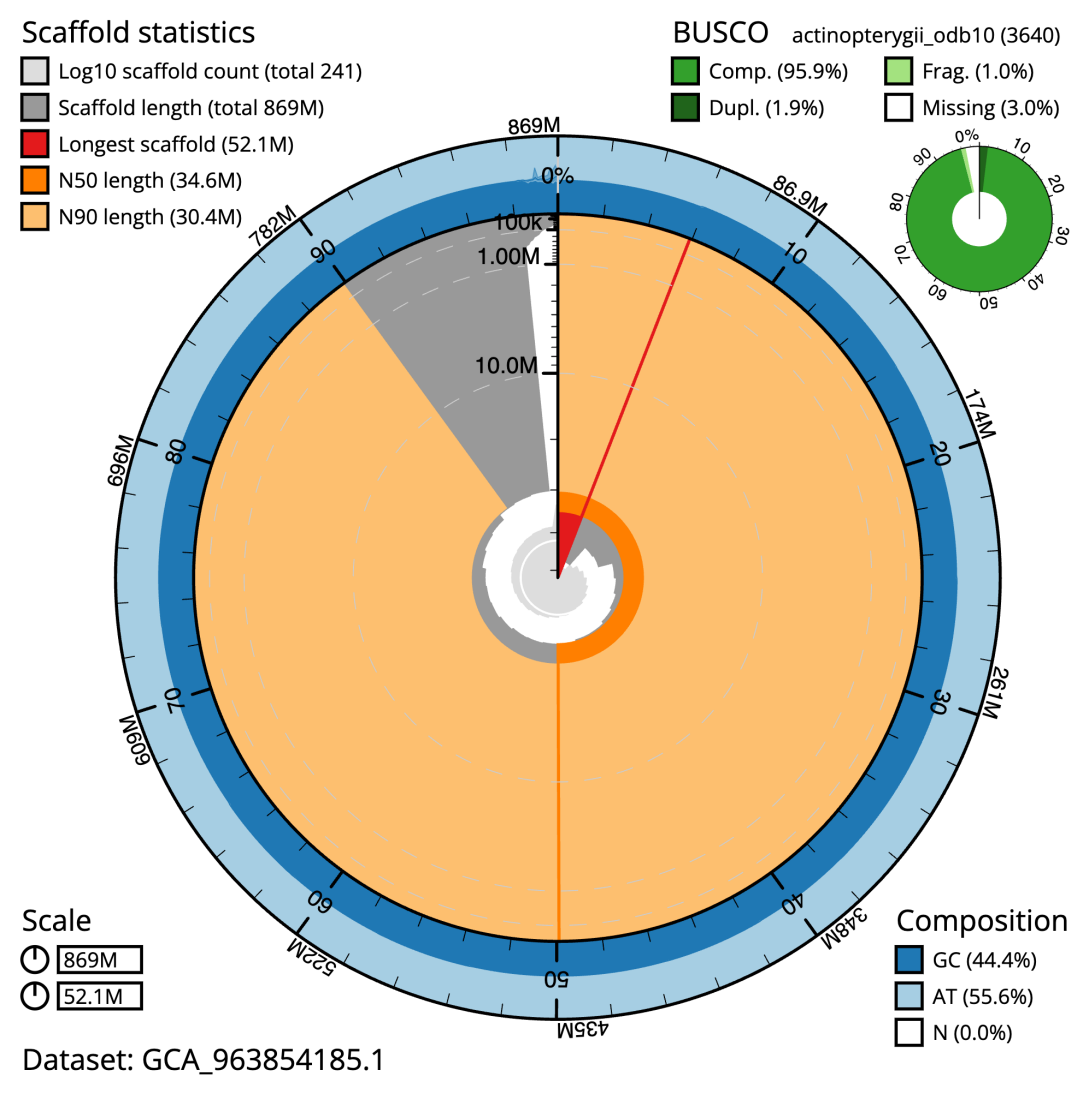
Genome assembly of *Sardina pilchardus*, fSarPil1.1: metrics. The BlobToolKit snail plot shows N50 metrics and BUSCO gene completeness. The main plot is divided into 1,000 bins around the circumference with each bin representing 0.1% of the 869,414,035 bp assembly. The distribution of scaffold lengths is shown in dark grey with the plot radius scaled to the longest scaffold present in the assembly (52,055,910 bp, shown in red). Orange and pale-orange arcs show the N50 and N90 scaffold lengths (34,617,450 and 30,366,703 bp), respectively. The pale grey spiral shows the cumulative scaffold count on a log scale with white scale lines showing successive orders of magnitude. The blue and pale-blue area around the outside of the plot shows the distribution of GC, AT and N percentages in the same bins as the inner plot. A summary of complete, fragmented, duplicated and missing BUSCO genes in the actinopterygii_odb10 set is shown in the top right. An interactive version of this figure is available at https://blobtoolkit.genomehubs.org/view/Sardina_pilchardus/dataset/GCA_963854185.1/snail.

**Figure 3.**
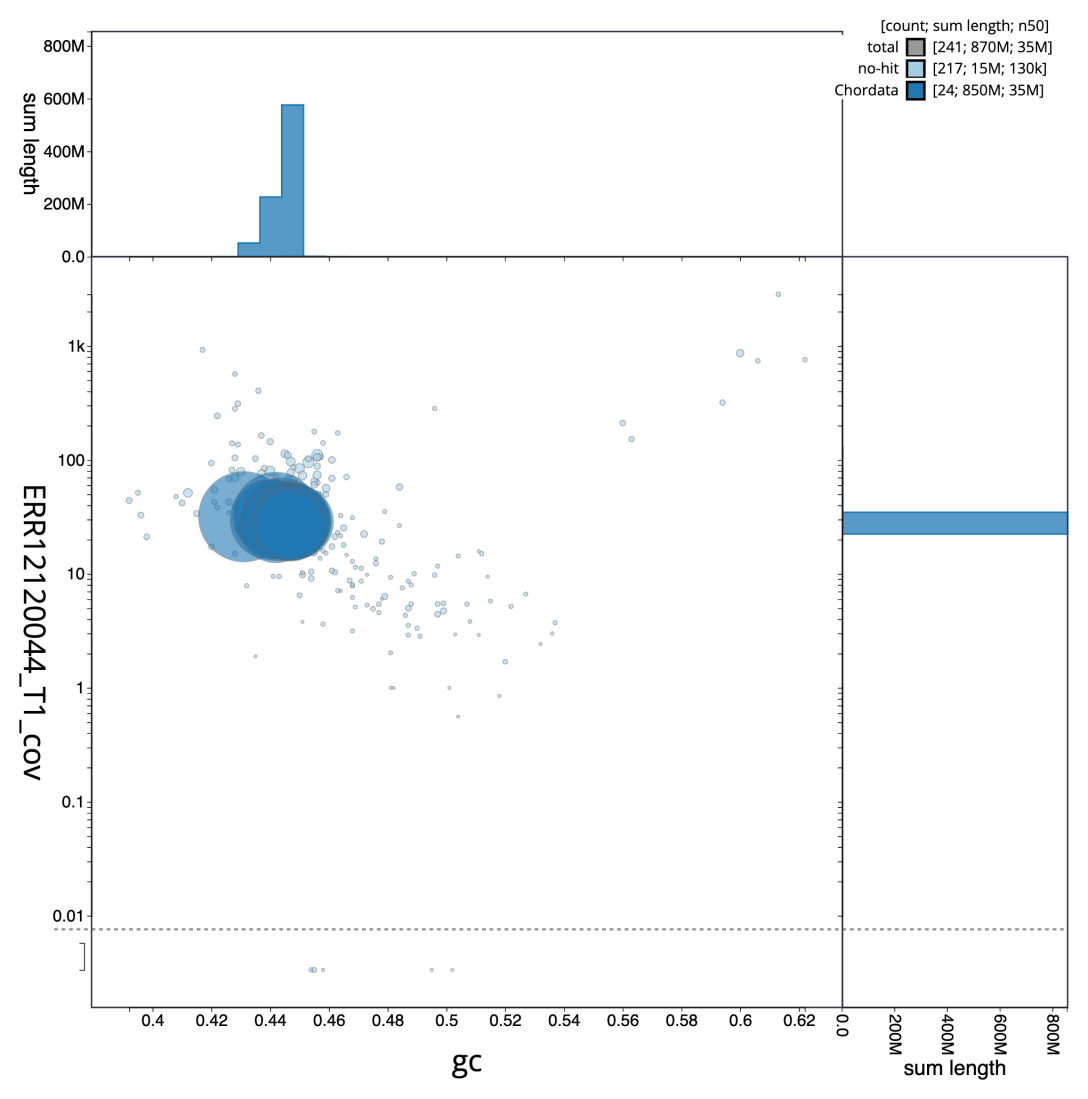
Genome assembly of *Sardina pilchardus*, fSarPil1.1: BlobToolKit GC-coverage plot. Sequences are coloured by phylum. Circles are sized in proportion to sequence length. Histograms show the distribution of sequence length sum along each axis. An interactive version of this figure is available at https://blobtoolkit.genomehubs.org/view/Sardina_pilchardus/dataset/GCA_963854185.1/blob.

**Figure 4.**
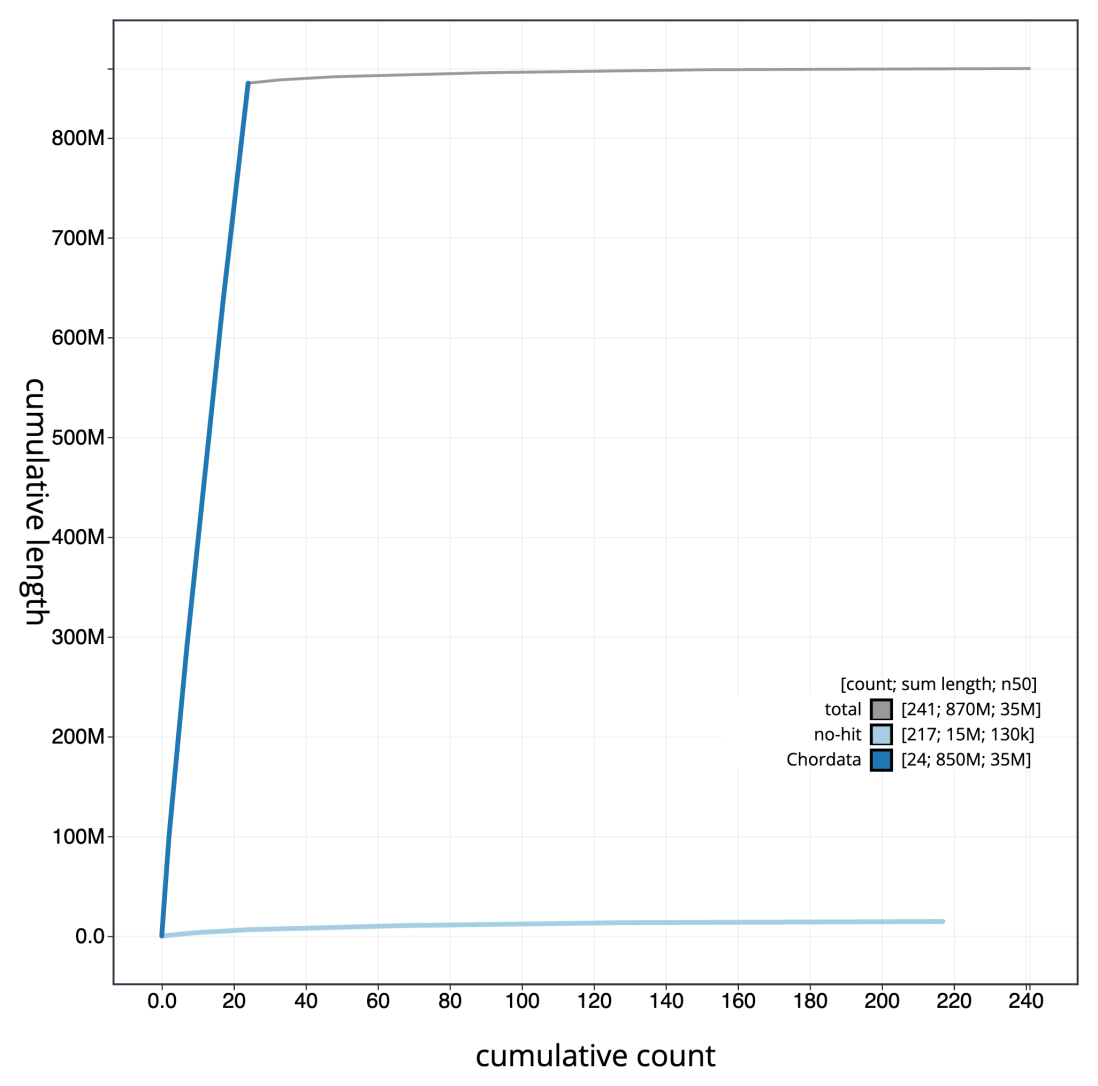
Genome assembly of *Sardina pilchardus* fSarPil1.1: BlobToolKit cumulative sequence plot. The grey line shows cumulative length for all sequences. Coloured lines show cumulative lengths of sequences assigned to each phylum using the buscogenes taxrule. An interactive version of this figure is available at https://blobtoolkit.genomehubs.org/view/Sardina_pilchardus/dataset/GCA_963854185.1/cumulative.

**Figure 5.**
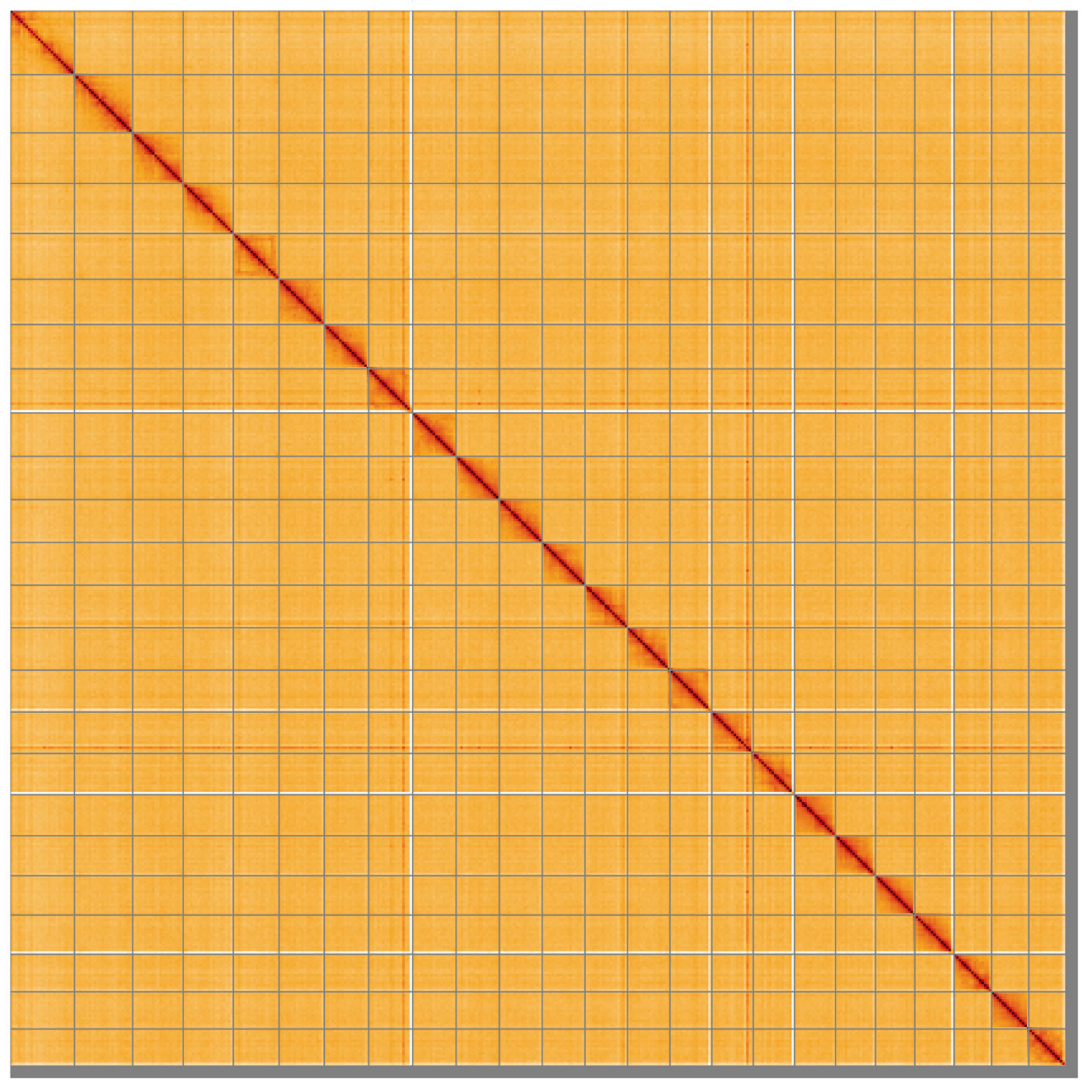
Genome assembly of *Sardina pilchardus* fSarPil1.1: Hi-C contact map of the fSarPil1.1 assembly, visualised using HiGlass. Chromosomes are shown in order of size from left to right and top to bottom. An interactive version of this figure may be viewed at https://genome-note-higlass.tol.sanger.ac.uk/l/?d=PbOTNKzwQwmKEkqs89tnRw.

Manual assembly curation corrected 328 missing joins or mis-joins, reducing the assembly length by 6.2% and the scaffold number by 21.5%, and increasing the scaffold N50 by 0.06%. The final assembly has a total length of 869.40 Mb in 240 sequence scaffolds with a scaffold N50 of 34.6 Mb ( Table 2). The total count of gaps in the scaffolds is 1,654. The snail plot in Figure 2 provides a summary of the assembly statistics, while the distribution of assembly scaffolds on GC proportion and coverage is shown in Figure 3. The cumulative assembly plot in Figure 4 shows curves for subsets of scaffolds assigned to different phyla. Most (98.33%) of the assembly sequence was assigned to 24 chromosomal-level scaffolds. Chromosome-scale scaffolds confirmed by the Hi-C data are named in order of size ( Figure 5; Table 3). There are large haplotypic inversions on the following chromosomes: Chromosome 5 from ~4.03–31.1Mb, Chromosome 8 from ~6.87–30.93Mb, Chromosome 9 from ~4.98–33.85Mb, Chromosome 15 from ~3.26–29.76, Chromosome 17, a double inversion from ~17.96–30.93. The alternate assembly has been assembled into chromosomes as a merged assembly of both haplotypes was used for curation. The mitochondrial genome was also assembled and can be found as a contig within the multifasta file of the genome submission.

**Table 2.** Genome assembly data for *Sardina pilchardus*, fSarPil1.1.

Genome assembly		
Assembly name	fSarPil1.1	
Assembly accession	GCA_963854185.1	
*Accession of alternate haplotype*	*GCA_963854175.1*	
Span (Mb)	869.40	
Number of contigs	1,895	
Contig N50 length (Mb)	1.1	
Number of scaffolds	240	
Scaffold N50 length (Mb)	34.6	
Longest scaffold (Mb)	52.06	
Assembly metrics *		*Benchmark*
Consensus quality (QV)	52.1	*≥ 40*
*k*-mer completeness	99.98%	*≥ 95%*
BUSCO **	C:95.4%[S:93.6%,D:1.8%], F:1.5%,M:3.1%,n:3,640	*S > 90%, D < 5%*
Percentage of assembly mapped to chromosomes	98.33%	*≥ 90%*
Sex chromosomes	Not identified	*localised homologous pairs*
Organelles	Mitochondrial genome: 17.57 kb	*complete single alleles*

* Assembly metric benchmarks are adapted from column VGP-2020 of “Table 1: Proposed standards and metrics for defining genome assembly quality” from Rhie *et al.* (2021). ** BUSCO scores based on the actinopterygii_odb10 BUSCO set using version 5.4.3. C = complete [S = single copy, D = duplicated], F = fragmented, M = missing, n = number of orthologues in comparison. A full set of BUSCO scores is available at https://blobtoolkit.genomehubs.org/view/Sardina_pilchardus/dataset/GCA_963854185.1/busco.

**Table 3.** Chromosomal pseudomolecules in the genome assembly of *Sardina pilchardus*, fSarPil1.

INSDC accession	Name	Length (Mb)	GC%
OY974086.1	1	52.06	43.0
OY974087.1	2	47.06	44.0
OY974088.1	3	40.94	44.5
OY974089.1	4	40.61	44.0
OY974090.1	5	37.04	44.0
OY974091.1	6	36.57	44.5
OY974092.1	7	36.05	44.5
OY974093.1	8	35.6	44.5
OY974094.1	9	35.11	44.5
OY974095.1	10	35.07	45.0
OY974096.1	11	34.7	44.5
OY974097.1	12	34.62	44.5
OY974098.1	13	34.46	45.0
OY974099.1	14	34.36	44.5
OY974100.1	15	33.95	45.0
OY974101.1	16	33.55	44.0
OY974102.1	17	33.39	44.5
OY974103.1	18	33.2	44.5
OY974104.1	19	32.36	44.5
OY974105.1	20	31.94	45.0
OY974106.1	21	31.68	44.5
OY974107.1	22	30.37	44.5
OY974108.1	23	30.18	44.5
OY974109.1	24	29.97	44.5
OY974110.1	MT	0.02	49.5

The estimated Quality Value (QV) of the final assembly is 52.1 with *k*-mer completeness of 99.98%, and the assembly has a BUSCO v5.4.3 completeness of 95.4% (single = 93.6%, duplicated = 1.8%), using the actinopterygii_odb10 reference set ( *n* = 3,640).

Metadata for specimens, BOLD barcode results, spectra estimates, sequencing runs, contaminants and pre-curation assembly statistics are given at https://links.tol.sanger.ac.uk/species/27697.

## Methods

### Sample acquisition

An adult *Sardina pilchardus* (specimen ID MBA-220324-003A, ToLID fSarPil1) was collected from an inshore fisheries net deployed from the RV Sepia) at Whitsand Bay, Cornwall, UK (latitude 50.33, longitude –4.24) on 2022-03-24. The sex of the specimen was not determined. The specimen was collected and identified by Kes Scott-Somme, Rachel Brittain, Patrick Adkins and Joanna Harley (Marine Biological Association). Tissue samples were preserved in liquid nitrogen.

The initial identification was verified by an additional DNA barcoding process according to the framework developed by Twyford *et al*. (2024). A small sample was dissected from the specimens and stored in ethanol, while the remaining parts of the specimen were shipped on dry ice to the Wellcome Sanger Institute (WSI). The tissue was lysed, the COI marker region was amplified by PCR, and amplicons were sequenced and compared to the BOLD database, confirming the species identification ( Crowley *et al.*, 2023). Following whole genome sequence generation, the relevant DNA barcode region was also used alongside the initial barcoding data for sample tracking at the WSI ( Twyford *et al.*, 2024). The standard operating procedures for Darwin Tree of Life barcoding have been deposited on protocols.io ( Beasley *et al.*, 2023).

### Nucleic acid extraction

The workflow for high molecular weight (HMW) DNA extraction at the Wellcome Sanger Institute (WSI) Tree of Life Core Laboratory includes a sequence of core procedures: sample preparation; sample homogenisation, DNA extraction, fragmentation, and clean-up. In sample preparation, the fSarPil1 sample was weighed and dissected on dry ice ( Jay *et al.*, 2023). Tissue from gills was homogenised using a PowerMasher II tissue disruptor ( Denton *et al.*, 2023a). HMW DNA was extracted using the Automated MagAttract v2 protocol ( Oatley *et al.*, 2023a). DNA was sheared into an average fragment size of 12–20 kb in a Megaruptor 3 system with speed setting 31 ( Bates *et al.*, 2023). Sheared DNA was purified by solid-phase reversible immobilisation ( Oatley *et al.*, 2023b): in brief, the method employs AMPure PB beads to eliminate shorter fragments and concentrate the DNA. The concentration of the sheared and purified DNA was assessed using a Nanodrop spectrophotometer and Qubit Fluorometer using the Qubit dsDNA High Sensitivity Assay kit. Fragment size distribution was evaluated by running the sample on the FemtoPulse system.

RNA was extracted from gill tissue of fSarPil1 in the Tree of Life Laboratory at the WSI using the RNA Extraction: Automated MagMax™ *mir*Vana protocol ( do Amaral *et al.*, 2023). The RNA concentration was assessed using a Nanodrop spectrophotometer and a Qubit Fluorometer using the Qubit RNA Broad-Range Assay kit. Analysis of the integrity of the RNA was done using the Agilent RNA 6000 Pico Kit and Eukaryotic Total RNA assay.

Protocols developed by the WSI Tree of Life laboratory are publicly available on protocols.io ( Denton *et al.*, 2023b).

### Sequencing

Pacific Biosciences HiFi circular consensus DNA sequencing libraries were constructed according to the manufacturers’ instructions. Poly(A) RNA-Seq libraries were constructed using the NEB Ultra II RNA Library Prep kit. DNA and RNA sequencing was performed by the Scientific Operations core at the WSI on Pacific Biosciences Revio (HiFi) and Illumina NovaSeq 6000 (RNA-Seq) instruments. Hi-C data were also generated from gill tissue of fSarPil1 using the Arima-HiC v2 kit. The Hi-C sequencing was performed using paired-end sequencing with a read length of 150 bp on the Illumina NovaSeq 6000 instrument.

### Genome assembly, curation and evaluation

***Assembly***

The original assembly of HiFi reads was performed using Hifiasm ( Cheng *et al.*, 2021) with the --primary option. Haplotypic duplications were identified and removed with purge_dups ( Guan *et al.*, 2020). Hi-C reads are further mapped with bwamem2 ( Vasimuddin *et al.*, 2019) to the primary contigs, which are further scaffolded using the provided Hi-C data ( Rao *et al.*, 2014) in YaHS ( Zhou *et al.*, 2023) using the --break option. Scaffolded assemblies are evaluated using Gfastats ( Formenti *et al.*, 2022), BUSCO ( Manni *et al.*, 2021) and MERQURY.FK ( Rhie *et al.*, 2020).

The mitochondrial genome was assembled using MitoHiFi ( Uliano-Silva *et al.*, 2023), which runs MitoFinder ( Allio *et al.*, 2020) and uses these annotations to select the final mitochondrial contig and to ensure the general quality of the sequence.

***Assembly curation***

The assembly was decontaminated using the Assembly Screen for Cobionts and Contaminants (ASCC) pipeline (article in preparation). Flat files and maps used in curation were generated in TreeVal ( Pointon *et al.*, 2023). Manual curation was primarily conducted using PretextView ( Harry, 2022), with additional insights provided by JBrowse2 ( Diesh *et al.*, 2023) and HiGlass ( Kerpedjiev *et al.*, 2018). Scaffolds were visually inspected and corrected as described by Howe *et al*. (2021). Any identified contamination, missed joins, and mis-joins were corrected, and duplicate sequences were tagged and removed. The entire process is documented at https://gitlab.com/wtsi-grit/rapid-curation (article in preparation).

***Evaluation of the final assembly***

The final assembly was post-processed and evaluated with the three Nextflow ( Di Tommaso *et al.*, 2017) DSL2 pipelines “sanger-tol/readmapping” ( Surana *et al.*, 2023a), “sanger-tol/genomenote” ( Surana *et al.*, 2023b), and “sanger-tol/blobtoolkit” ( Muffato *et al.*, 2024). The pipeline sanger-tol/readmapping aligns the Hi-C reads with bwa-mem2 ( Vasimuddin *et al.*, 2019) and combines the alignment files with SAMtools ( Danecek *et al.*, 2021). The sanger-tol/genomenote pipeline transforms the Hi-C alignments into a contact map with BEDTools ( Quinlan & Hall, 2010) and the Cooler tool suite ( Abdennur & Mirny, 2020), which is then visualised with HiGlass ( Kerpedjiev *et al.*, 2018). It also provides statistics about the assembly with the NCBI datasets ( Sayers *et al.*, 2024) report, computes *k*-mer completeness and QV consensus quality values with FastK and MERQURY.FK, and a completeness assessment with BUSCO ( Manni *et al.*, 2021).

The sanger-tol/blobtoolkit pipeline is a Nextflow port of the previous Snakemake Blobtoolkit pipeline ( Challis *et al.*, 2020). It aligns the PacBio reads with SAMtools and minimap2 ( Li, 2018) and generates coverage tracks for regions of fixed size. In parallel, it queries the GoaT database ( Challis *et al.*, 2023) to identify all matching BUSCO lineages to run BUSCO ( Manni *et al.*, 2021). For the three domain-level BUSCO lineage, the pipeline aligns the BUSCO genes to the Uniprot Reference Proteomes database ( Bateman *et al.*, 2023) with DIAMOND ( Buchfink *et al.*, 2021) blastp. The genome is also split into chunks according to the density of the BUSCO genes from the closest taxonomically lineage, and each chunk is aligned to the Uniprot Reference Proteomes database with DIAMOND blastx. Genome sequences that have no hit are then chunked with seqtk and aligned to the NT database with blastn ( Altschul *et al.*, 1990). All those outputs are combined with the blobtools suite into a blobdir for visualisation.

The genome assembly and evaluation pipelines were developed using the nf-core tooling ( Ewels *et al.*, 2020), use MultiQC ( Ewels *et al.*, 2016), and make extensive use of the Conda package manager, the Bioconda initiative ( Grüning *et al.*, 2018), the Biocontainers infrastructure ( da Veiga Leprevost *et al.*, 2017), and the Docker ( Merkel, 2014) and Singularity ( Kurtzer *et al.*, 2017) containerisation solutions. Table 4 contains a list of relevant software tool versions and sources.

**Table 4.** Software tools: versions and sources.

Software tool	Version	Source
BEDTools	2.30.0	https://github.com/arq5x/bedtools2
BLAST	2.14.0	ftp://ftp.ncbi.nlm.nih.gov/blast/executables/blast+/
BlobToolKit	4.3.7	https://github.com/blobtoolkit/blobtoolkit
BUSCO	5.4.3 and 5.5.0	https://gitlab.com/ezlab/busco
bwa-mem2	2.2.1	https://github.com/bwa-mem2/bwa-mem2
Cooler	0.8.11	https://github.com/open2c/cooler
DIAMOND	2.1.8	https://github.com/bbuchfink/diamond
fasta_windows	0.2.4	https://github.com/tolkit/fasta_windows
FastK	427104ea91c78c3b8b8b49f1a7d6bbeaa869ba1c	https://github.com/thegenemyers/FASTK
Gfastats	1.3.6	https://github.com/vgl-hub/gfastats
GoaT CLI	0.2.5	https://github.com/genomehubs/goat-cli
Hifiasm	0.19.5-r587	https://github.com/chhylp123/hifiasm
HiGlass	44086069ee7d4d3f6f3f0012569789ec138f42b84a a44357826c0b6753eb28de	https://github.com/higlass/higlass
Merqury.FK	d00d98157618f4e8d1a9190026b19b471055b22e	https://github.com/thegenemyers/MERQURY.FK
MitoHiFi	3	https://github.com/marcelauliano/MitoHiFi
MultiQC	1.14, 1.17, and 1.18	https://github.com/MultiQC/MultiQC
NCBI Datasets	15.12.0	https://github.com/ncbi/datasets
Nextflow	23.04.0-5857	https://github.com/nextflow-io/nextflow
PretextView	0.2	https://github.com/sanger-tol/PretextView
purge_dups	1.2.5	https://github.com/dfguan/purge_dups
samtools	1.16.1, 1.17, and 1.18	https://github.com/samtools/samtools
sanger-tol/ascc	-	https://github.com/sanger-tol/ascc
sanger-tol/ genomenote	1.1.1	https://github.com/sanger-tol/genomenote
sanger-tol/ readmapping	1.2.1	https://github.com/sanger-tol/readmapping
Seqtk	1.3	https://github.com/lh3/seqtk
Singularity	3.9.0	https://github.com/sylabs/singularity
TreeVal	1.0.0	https://github.com/sanger-tol/treeval
YaHS	1.2a.2	https://github.com/c-zhou/yahs

### Wellcome Sanger Institute – Legal and Governance 

The materials that have contributed to this genome note have been supplied by a Darwin Tree of Life Partner. The submission of materials by a Darwin Tree of Life Partner is subject to the **‘Darwin Tree of Life Project Sampling Code of Practice’**, which can be found in full on the Darwin Tree of Life website here. By agreeing with and signing up to the Sampling Code of Practice, the Darwin Tree of Life Partner agrees they will meet the legal and ethical requirements and standards set out within this document in respect of all samples acquired for, and supplied to, the Darwin Tree of Life Project.

Further, the Wellcome Sanger Institute employs a process whereby due diligence is carried out proportionate to the nature of the materials themselves, and the circumstances under which they have been/are to be collected and provided for use. The purpose of this is to address and mitigate any potential legal and/or ethical implications of receipt and use of the materials as part of the research project, and to ensure that in doing so we align with best practice wherever possible. The overarching areas of consideration are:

•     Ethical review of provenance and sourcing of the material

•     Legality of collection, transfer and use (national and international) 

Each transfer of samples is further undertaken according to a Research Collaboration Agreement or Material Transfer Agreement entered into by the Darwin Tree of Life Partner, Genome Research Limited (operating as the Wellcome Sanger Institute), and in some circumstances other Darwin Tree of Life collaborators.

## Data Availability

European Nucleotide Archive: *Sardina pilchardus* (sardine). Accession number PRJEB67417; https://identifiers.org/ena.embl/PRJEB67417 ( Wellcome Sanger Institute, 2023). The genome sequence is released openly for reuse. The *Sardina pilchardus* genome sequencing initiative is part of the Darwin Tree of Life (DToL) project. All raw sequence data and the assembly have been deposited in INSDC databases. The genome will be annotated using available RNA-Seq data and presented through the Ensembl pipeline at the European Bioinformatics Institute. Raw data and assembly accession identifiers are reported in Table 1 and Table 2.

## Author information

Members of the Marine Biological Association Genome Acquisition Lab are listed here: https://doi.org/10.5281/zenodo.8382513.

Members of the Darwin Tree of Life Barcoding collective are listed here: https://doi.org/10.5281/zenodo.4893703.

Members of the Wellcome Sanger Institute Tree of Life Management, Samples and Laboratory team are listed here: https://doi.org/10.5281/zenodo.10066175.

Members of Wellcome Sanger Institute Scientific Operations: Sequencing Operations are listed here: https://doi.org/10.5281/zenodo.10043364.

Members of the Wellcome Sanger Institute Tree of Life Core Informatics team are listed here: https://doi.org/10.5281/zenodo.10066637.

Members of the Tree of Life Core Informatics collective are listed here: https://doi.org/10.5281/zenodo.5013541.

Members of the Darwin Tree of Life Consortium are listed here: https://doi.org/10.5281/zenodo.4783558.
