# Supplementary material for: The genome sequence of the sardine, Sardina pilchardus (Walbaum, 1792)
Source: Wellcome Open Res. 2024 Nov 4;9:434. Originally published 2024 Aug 7. [Version 2] doi: 10.12688/wellcomeopenres.22826.2 (PMC11582392; doi:10.12688/wellcomeopenres.22826.2)
Supplement: Peer review history

# Reviewer report: Reviewer response for version 2

**DOI:** 10.21956/wellcomeopenres.25736.r111036

**Published:** 2024-12-09

**Authors:** Grigorios Krey

**Affiliations:** 1 Hellenic Agricultural Organization-DIMITRA (ELGO-DIMITRA), Nea Peramos, Greece

The report of Brittain et al., on the genome sequence of the European sardine ( *Sardina pilchardus*), represents a high-quality assembly at the chromosome level (>98% complete). This genome sequence (annotated by the NCBI Eukaryotic Genome Annotation Pipeline) has been publicly accessible, for several months now, providing the data for comparative analyses studies, or, as the authors state, for population, ecological, and conservation studies concerning this particular species.

 This work has been preceded by two draft genome assemblies (scaffold level) generated independently by Machado et al. (2018) and Louro et al. (2019). The authors may want to consider mentioning these two studies in their present report.

 At the bottom of the first paragraph in the Background section, the reference should be corrected to (Fischer, 1973).

Are sufficient details of methods and materials provided to allow replication by others?

Yes

Is the rationale for creating the dataset(s) clearly described?

Yes

Are the datasets clearly presented in a useable and accessible format?

Yes

Are the protocols appropriate and is the work technically sound?

Yes

Reviewer Expertise:

Biochemistry/Molecular Biology

I confirm that I have read this submission and believe that I have an appropriate level of expertise to confirm that it is of an acceptable scientific standard.

# Reviewer report: Reviewer response for version 2

**DOI:** 10.21956/wellcomeopenres.25736.r111033

**Published:** 2024-12-07

**Authors:** Ricardo Pérez Enriquez

**Affiliations:** 1 Centro de Investigaciones Biológicas del Noroeste, El Comitán, Mexico

The MS describes the process of the genome assembly of the sardine *Sardina pilchardus, *an economically relevant fishery species. The sequencing information used for the assembly was based on several platforms starting with PacBio and single-molecule HiFi long reads, and complemented with Hi-C, and Illumina RNA-Seq data. This provides a strong background for the assembly, with appropriate filtering, assembling, curation, and evaluation protocols. A comprehensive list of the scripts used and maintained in github allow for others to follow similar paths for other genome assemblies. The data are also available for further use and exploration.

 Nevertheless, attending some minor issues may help to improve the MS:

- The abstract misses some detailed information on the findings (e.g. N50, longest scaffold, BUSCO results, relevance of the results), that would be of benefit for readers.
- Background on previous genome characterization of the species is needed. Is there any genome assembly reported for the species or taxonomic related species?
- In the text “Scaffolded assemblies are evaluated using Gfastats (Formenti et al., 2022), BUSCO (Manni et al., 2021) and MERQURY.FK (Rhie et al., 2020).” Please provide details on what does each software evaluates.
- Is it possible that the “Assembly Screen for Cobionts and Contaminants (ASCC) pipeline” would made available at github?
- I don’t recommend using the text “article in preparation” (it is used twice).
- Homogenize the terminology of the program bwa-mem2.
- At the end of the “Genome sequence report” section where the estimated Quality Value (QV) parameters are described, how these numbers compare with other species? This would also derive in some sort of conclusion about the relevance of the study.

Are sufficient details of methods and materials provided to allow replication by others?

Yes

Is the rationale for creating the dataset(s) clearly described?

Yes

Are the datasets clearly presented in a useable and accessible format?

Yes

Are the protocols appropriate and is the work technically sound?

Yes

Reviewer Expertise:

Population genetics and genomics of marine species.

I confirm that I have read this submission and believe that I have an appropriate level of expertise to confirm that it is of an acceptable scientific standard.

# Reviewer report: Reviewer response for version 2

**DOI:** 10.21956/wellcomeopenres.25736.r109643

**Published:** 2024-11-26

**Authors:** Shigehiro Kuraku

**Affiliations:** 1 National Institute of Genetics, Shizuoka, Japan

I have confirmed the revision and accept this manuscript.

Are sufficient details of methods and materials provided to allow replication by others?

No

Is the rationale for creating the dataset(s) clearly described?

Yes

Are the datasets clearly presented in a useable and accessible format?

Yes

Are the protocols appropriate and is the work technically sound?

Yes

Reviewer Expertise:

NA

I confirm that I have read this submission and believe that I have an appropriate level of expertise to confirm that it is of an acceptable scientific standard.

# Reviewer report: Reviewer response for version 2

**DOI:** 10.21956/wellcomeopenres.25736.r111032

**Published:** 2024-11-21

**Authors:** Satoshi Kawato

**Affiliations:** 1 Tokyo University of Marine Science and Technology, Tokyo, Japan

This work presents a chromosome-level genome assembly of the European sardine. The assembly quality is high, and the methods (both wet and dry) are well documented. The concerns I would have raised were already addressed in the first round of review. I would like to congratulate the authors on their work.

Are sufficient details of methods and materials provided to allow replication by others?

Yes

Is the rationale for creating the dataset(s) clearly described?

Yes

Are the datasets clearly presented in a useable and accessible format?

Yes

Are the protocols appropriate and is the work technically sound?

Yes

Reviewer Expertise:

Aquaculture;Genomics;Microbiology

I confirm that I have read this submission and believe that I have an appropriate level of expertise to confirm that it is of an acceptable scientific standard.

# Reviewer report: Reviewer response for version 1

**DOI:** 10.21956/wellcomeopenres.25133.r95666

**Published:** 2024-09-24

**Authors:** Shigehiro Kuraku

**Affiliations:** 1 National Institute of Genetics, Shizuoka, Japan

The authors report the whole genome assembly of the European Sardine, while I find quite well-assembled.

 The authors should not leave the sex of the animal used unmentioned and need to state if it is female, male or unknown.

 I wonder why the authors do not use the program compleasm (with a possibly enhanced sensitivity in exon detection) to evaluate the completeness of the genome assembly obtained.

Are sufficient details of methods and materials provided to allow replication by others?

No

Is the rationale for creating the dataset(s) clearly described?

Yes

Are the datasets clearly presented in a useable and accessible format?

Yes

Are the protocols appropriate and is the work technically sound?

Yes

Reviewer Expertise:

genome informatics

I confirm that I have read this submission and believe that I have an appropriate level of expertise to confirm that it is of an acceptable scientific standard, however I have significant reservations, as outlined above.

# Response

**Published:** 2024-10-26

**Authors:** Tree of Life Team Sanger

**Affiliations:** Wellcome Trust Sanger Institute, UK

Thank you for your review of this data note. 

 We have added text to clarify that the sex of this specimen was not determined.

# Reviewer report: Reviewer response for version 1

**DOI:** 10.21956/wellcomeopenres.25133.r95671

**Published:** 2024-09-16

**Authors:** Leif Andersson

**Affiliations:** 1 Medical Biochemistry and Microbiology, Uppsala University, Uppsala, Sweden; 2 Department of Veterinary Integrative Biosciences, Texas A&M University, College Station, Texas, USA

This Data Note describes the development of a high-quality assembly of the European sardine genome. The assembly meets high benchmark scores for high quality assemblies. I have only some minor suggestions for changes:

 1. Please specify where the fish was caught and sex of the fish, if known, in the beginning of the Genome sequence report section. Info about location is provided in the Methods section but it does not hurt if this info is repeated. Information about sex is not provided as far as I can see.

 2. Large haplotype inversions were noted on 5 chromosomes. It would be useful to know if you think this could be assembly artifacts or if the data strongly support the interpretation that the reference individual was heterozygous for these 5 inversions.

 3. Sample acquisition: There is a typo in the 2nd paragraph, change “wass” to was”

Are sufficient details of methods and materials provided to allow replication by others?

Yes

Is the rationale for creating the dataset(s) clearly described?

Yes

Are the datasets clearly presented in a useable and accessible format?

Yes

Are the protocols appropriate and is the work technically sound?

Yes

Reviewer Expertise:

Evolutionary genomics

I confirm that I have read this submission and believe that I have an appropriate level of expertise to confirm that it is of an acceptable scientific standard.

# Response

**Published:** 2024-10-26

**Authors:** Tree of Life Team Sanger

**Affiliations:** Wellcome Trust Sanger Institute, UK

Thank you for reviewing this data note. We have the following responses to your comments:

 1. The sex of this specimen was not determined on collection. There is little external sexual dimorphism in *Sardina pilchardus*.

 2. Haplotypic inversions were observed on several chromosomes. To thoroughly investigate these inversions, we combined both haplotypes during the curation process. This approach allowed us to integrate information from both alleles, providing a more comprehensive and accurate representation of the genomic regions in question. By analysing the combined data, we were able to clarify the structure of these inversions and ensure that they are accurately reflected in the assembly. We are confident that these are not assembly artefacts based on close investigation of the Hi-C map in PretextView and HiGlass. 3. We have corrected the typo in the Sample acquisition section.
